# Letter to the Editor. Re: “[An extensive dataset of handwritten central Kurdish isolated characters by R.M. Ahmed, T.A. Rashid, P. Fatah, A. Alsadoon & S. Mirjalili, Data in Brief, 2021, 39, 107479]”

**DOI:** 10.1016/j.dib.2023.109748

**Published:** 2023-10-31

**Authors:** Peshraw Ahmed Abdalla, Bashdar Abdalrahman Mohammed

**Affiliations:** Computer Science Department, College of Science, University of Halabja, Kurdistan Region, Halabja 46018, Iraq

To the editors:

We are writing in reference to the paper titled “An Extensive Dataset of Handwritten Central Kurdish Isolated Characters” [1]. Concerning the dataset URLs, the dataset is publicly accessible via the following URL: https://data.mendeley.com/datasets/f8z9jts5nb/2. It is important to emphasize that this URL is the correct reference for the dataset. Despite an alternative URL (http://dx.doi.org/10.17632/f8z9jts5nb.1) being listed in the Specification Table, we have rigorously confirmed that both versions of the dataset are identical in terms of data content and image numbers. Therefore, we have selected https://data.mendeley.com/datasets/f8z9jts5nb/2 as the preferred URL for accessibility and clarity. While the authors provided a huge dataset for Kurdish language (Sorani). We acknowledge this work, but we would like to highlight two major issues that certainly change the actual numbers and image proportions, these discrepancies and errors that need correction in the published paper.

1 Total Number of Images: In the paper, it is mentioned that the dataset contains a total of 40,940 images. However, after a careful review, it is evident that the dataset actually contains 40,826 images. This discrepancy in the reported number of images needs to be corrected, as it affects the accuracy of the dataset description.
2 Errors in Tables: There are errors in the proportions and percentages presented in the tables. To illustrate these errors, we have clearly shown the discrepancies in the class numbers. These inaccuracies may lead to misinterpretations of the dataset's contents and characteristics.

As an aid to the correction process, we prepared Table 1 which lists all the incorrect image numbers alongside their actual values. ensuring that the dataset's description, numbers, and percentages accurately reflect the content. This correction will be valuable to researchers and readers who rely on the accuracy of the dataset for their work. In terms of the incorrect image number, the letters (ژ, س, and ش) are affected as explained in Table 1, these incorrect classes also affected the total percentage of other classes.

**Table 1:** The actual and incorrect number and percentage of the collected letters.

ID	Letter	Incorrect number of images	Actual number of images	Incorrect percentage	Actual Percentage
1	ئـ	1134	1134	**2.77%**	**2.78%**
2	ا	1134	1134	**2.77%**	**2.78%**
3	ب	1134	1134	**2.77%**	**2.78%**
4	پ	1008	1008	**2.46%**	**2.47%**
5	ت	1134	1134	**2.77%**	**2.78%**
6	ج	1134	1134	**2.77%**	**2.78%**
7	چ	1260	1260	**3.08%**	**3.09%**
8	ح	1260	1260	**3.08%**	**3.09%**
9	خ	1134	1134	**2.77%**	**2.78%**
10	د	1134	1134	**2.77%**	**2.78%**
11	ر	1134	1134	**2.77%**	**2.78%**
12	ڕ	1134	1134	**2.77%**	**2.78%**
13	ز	1512	1512	**3.69%**	**3.70%**
14	**ژ**	**1123**	**1134**	**2.74%**	**2.78%**
15	**س**	**1107**	**1108**	**2.70%**	**2.71%**
16	**ش**	**1134**	**1008**	**2.77%**	**2.47%**
17	ع	1260	1260	**3.08%**	**3.09%**
18	غ	1134	1134	**2.77%**	**2.78%**
19	ف	1134	1134	**2.77%**	**2.78%**
20	ڤ	1134	1134	**2.77%**	**2.78%**
21	ق	1260	1260	**3.08%**	**3.09%**
22	ک	1386	1386	**3.39%**	**3.39%**
23	ك	883	883	**2.16%**	**2.16%**
24	گ	1134	1134	**2.77%**	**2.78%**
25	ل	1134	1134	**2.77%**	**2.78%**
26	ڵ	1134	1134	**2.77%**	**2.78%**
27	م	1386	1386	**3.39%**	**3.39%**
28	ن	1161	1161	**2.84%**	**2.84%**
29	هـ	1008	1008	**2.46%**	**2.47%**
30	ە	1512	1512	**3.69%**	**3.70%**
31	و	1134	1134	**2.77%**	**2.78%**
32	ۆ	1134	1134	**2.77%**	**2.78%**
33	وو	1134	1134	**2.77%**	**2.78%**
34	ی	1134	1134	**2.77%**	**2.78%**
35	ێ	1134	1134	**2.77%**	**2.78%**
		**40940**	**40826**	100%	100.00%

According to the authors, there were ten sets of forms, each set with 35 forms for 35 different letters. As explained in Table 2 the number of images for the incorrect sets for several characters including (ژ، س، ش) since the total number of the sets don't match the actual number of images for each Kurdish (Sorani) character. Also, it affected the total number of images for all 35 class characters.

**Table 2:** Incorrect image number of sets used for data collection.

ID	Letter	Set 1	Set 2	Set 3	Set 4	Set 5	Set 6	Set 7	Set 8	Set 9	Set 10	Incorrect Total
14	ژ	126	0	126	126	126	0	0	126	126	367	1123
15	س	126	126	126	99	126	126	126	126	126	0	1107
16	ش	126	0	126	126	126	126	126	126	126	126	1134
	**Incorrect** **Total for all 35 class**	**4410**	**3528**	**4410**	**4158**	**4158**	**4031**	**3905**	**4536**	**4284**	**3520**	**40940**

Our aim is to address these issues by publishing an erratum or correction to the paper, ensuring that the dataset's description, numbers, and percentages accurately reflect the content. This correction will be valuable to researchers and readers who rely on the accuracy of the dataset for their work.

## Ethics Statement

In accordance with the guidelines of Data in Brief, we confirm that this letter to the editor does not present original research and, therefore, does not involve human subjects, animal experiments, or data collected from social media platforms. Consequently, there are no ethical approval, informed consent, or ethical considerations related to these aspects relevant to this correspondence.

## Funding

None.

## CRediT authorship contribution statement

**Peshraw Ahmed Abdalla:** Supervision, Conceptualization, Project administration, Writing – original draft, Writing – review & editing. **Bashdar Abdalrahman Mohammed:** Validation, Writing – review & editing.
